# Using *Guasha *to treat musculoskeletal pain: A systematic review of controlled clinical trials

**DOI:** 10.1186/1749-8546-5-5

**Published:** 2010-01-29

**Authors:** Myeong Soo Lee, Tae-Young Choi, Jong-In Kim, Sun-Mi Choi

**Affiliations:** 1 Acupuncture, Moxibustion and Meridian Research Center, Division of Standard Research, Korea Institute of Oriental Medicine, Daejeon 305-811, South Korea

## Abstract

**Background:**

*Guasha *is a therapeutic method for pain management using tools to scrape or rub the surface of the body to relieve blood stagnation. This study aims to systematically review the controlled clinical trials on the effectiveness of using *Guasha *to treat musculoskeletal pain.

**Methods:**

We searched 11 databases (without language restrictions): MEDLINE, Allied and Complementary Medicine (AMED), EMBASE, Cumulative Index to Nursing and Allied Health Literature (CINAHL), Korean Studies Information (KSI), DBPIA, Korea Institute of Science and Technology Information (KISTI), KoreaMed, Research Information Service System (RISS), China National Knowledge Infrastructure (CNKI) and the Cochrane Library. The search strategy was *Guasha *(OR scraping) AND pain. Risk of bias was assessed with the Cochrane criteria (i.e. sequence generation, blinding, incomplete outcome measures and allocation concealment).

**Results:**

Five randomized controlled trials (RCTs) and two controlled clinical trials (CCTs) were included in the present study. Two RCTs compared *Guasha *with acupuncture in terms of effectiveness, while the other trials compared *Guasha *with no treatment (1 trial), acupuncture (4 trials), herbal injection (1 trial) and massage or electric current therapy (1 trial). While two RCTs suggested favorable effects of *Guasha *on pain reduction and response rate, the quality of these RCTs was poor. One CCT reported beneficial effects of *Guasha *on musculoskeletal pain but had low methodological quality.

**Conclusion:**

Current evidence is insufficient to show that *Guasha *is effective in pain management. Further RCTs are warranted and methodological quality should be improved.

## Background

*Guasha *was defined as a therapeutic modality that uses several tools to scrape or rub the surface of the body to relieve blood (*Xue*) stagnation. *Guasha *is used for pain relief in Chinese medicine. Tools for *Guasha *including a Chinese soup spoon, an edge-worn coin, a slice of water-buffalo horn, a cow rib, honed jade and a simple metal cap with a smooth round lip with oil or water are used in *Guasha *to scrape or rub the skin to relieve blood stagnation at the body surface [1]. *Guasha *is also used to treat common cold, flu, respiratory problems and musculoskeletal (MS) pain [2].

There are three possible mechanisms of using *Guasha *to relieve MS pain: (1) *Guasha *increases local microcirculation thereby decreasing distal myalgia [1]; (2) pain is reduced through stimulating the serotonergic, noradrenergic and opioid systems; (3) *Guasha *minimizes the direct effects of pain at nociceptors, their surroundings and the interconnections within the spinal cord [3]. However, none of these theories can be established before actual effectiveness of *Guasha *is demonstrated.

To date, no systematic review is available to evaluate the effectiveness of using *Guasha *to treat MS pain. The present systematic review aims to critically evaluate the results of controlled clinical trials on the effectiveness of using *Guasha *to treat MS pain.

## Methods

### Data sources

The following databases were searched between their inception and July 2009: MEDLINE (1969), Allied and Complementary Medicine (AMED) (1995), EMBASE (1966), Cumulative Index to Nursing and Allied Health Literature (CINAHL) (1981), Korean Studies Information (KSI) (1966), DBPIA (1966), Korea Institute of Science and Technology Information (KISTI) (1959), KoreaMed (1959), Research Information Service System (RISS) (1959), China National Knowledge Infrastructure (CNKI) (1974) and the Cochrane Library (Issue 3, 2009). The search strategy was *Guasha *(OR scraping) AND pain. Korean and Chinese terms for *Guasha *AND pain were used when searching the Korean and Chinese databases. We also searched in the journals Focus on Alternative and Complementary Therapies (FACT) and Research in Complementary Medicine (Forschende Komplementarmedizin) electronically published between 1994 and July 2009. In addition, the references in all retrieved articles as well as our department files were searched.

### Study selection

We included all controlled clinical trials on using *Guasha *to treat patients (regardless gender or age) diagnosed with MS pain. Trials published as journal articles, dissertations and abstracts were eligible. We excluded the trials that compared one type of *Guasha *with another. Trials with *Guasha *as a part of a complex intervention were also excluded. No language restrictions were imposed.

### Data extraction and quality assessment

Hard copies of all articles included in the study were read in full independently by two authors (TYC, JIK). Data from the articles were validated and extracted according to pre-defined criteria (Table 1).

**Table 1:** Summary of controlled clinical studies of *Guasha *for musculoskeletal pain conditions

First author (year)	Design/sample size Conditions	Intervention group (regime)	Main outcomes	Results
Tang (2008) [5]	RCT/120 Fibromyalgia syndrome	(A) *Guasha *(n.r., once per 3 days, 5 times total, n = 60) (B) AT (30 min, once daily, 15 times, n = 60)	1) VAS (100 mm) 2) Number of pain points 3) Response rate	1) MD, -9.5, 95% CIs (-14.5 to -4.5) P < 0.0002 in favor of A 2) MD, -5.0, 95% CIs (-6.5 to -3.5), P < 0.0001 in favor of A 3) (A) 29/16/10/8; (B) 10/8/12/20 Improved 1.3 [0.94, 1.13], P = 0.01 Recovery 2.9 [1.55, 5.41], P = 0.0008
Chen (1995) [6]	RCT/90 Neck stiffness	(A) *Guasha *(20 min, once per 3~7 days, n.r., n = 30) (B) Massage (10 min, n = 30) (C) Electric current therapy (10 min, n = 30)	Response rate	(A) 27/1/2/0; (B) 27/2/1/0; (C) 28/1/1/0 NS NS
Ma (2003) [7]	RCT/50 Cervical spondylosis	(A) *Guasha *(1 session = n.r., once per 2 days, total 10 times, n = 15) (B) Herbal injection (once daily, total 20 times, n = 35)	Response rate	(A) 0/7/6/2; (B) 0/25/7/1 Improved 0.92 [0.74, 1.14], NS Recovery N/A
Wu (1996) [8]	RCT/100 Cervical spondylosis	(A) *Guasha *(n.r., once per 2 days, total 10 times, n = 72) (B) No treatment (n = 28)	Response rate	(A) 39/0/28/5; (B) 14/0/8/6 Improved 1.18 [0.97, 1.45], NS^†^ Recovery 1.08 [0.71, 1.66], NS
Li (1996) [9]	RCT/60 Scapulohumeral periarthritis	(A) *Guasha *(n.r., once per 4~5 days, total 5 times, n = 30) (B) AT (20 min, once daily, total 10 times, n = 30)	Response rate	(A) 18/8/4/0; (B) 10/10/8/2 Improved 1.07 [0.96, 1.20], NS^†^ Recovery 1.8 [1.00, 3.25], P = 0.05
Guo (1995) [10]	CCT/76 Cervical spondylosis	(A) *Guasha *(1 session = 20 min, once per 3 days, total 10 times, 2 session, n = 38) (B) AT (1 session = 30 min, once per 2 days, total 10 times, 2 session, n = 38)	Response rate	(A) 29/6/2/1;(B) 19/7/9/3 Improved 1.06 [0.95, 1.18], NS^†^ Recovery 1.53 [1.06, 2.20], P = 0.02
Wang (2004) [11]	CCT/240 Lumbar disc herniation	(A) *Guasha *(1 session = n.r., once per 7 days, total 3 times, 3 session, n = 160) (B) AT plus moxa (n = 80)	Response rate	(A) 32/69/45/14;(B) 11/27/33/9 Improved 1.03 [0.94, 1.13], NS^†^ Recovery 1.45 [0.77, 2.73], NS

n.r.: not reported; NS: no significance; AT: acupuncture; RCT: randomized controlled trial; CCT: controlled clinical trial; VAS: visual analog scale

Response rate was divided to four categories: (1) recovery, (2) marked improvement, (3) improvement and (4) no change

^†^We re-calculated the significance with RevMan for two categories: improved cases and recovery cases of each group.

The original authors reported a statistical significance for these studies (P < 0.05).

The Cochrane classification with four criteria (i.e. sequence generation, blinding, incomplete outcome measures and allocation concealment) was used to assess the risk of bias [4]. As it is difficult to blind *Guasha *therapists, we assessed the blinding of patient and assessor separately. A point was given for assessor blinding if pain was assessed by another person (who was unaware of the group assignment). Disagreements were resolved between the two authors (TYC, JIK) through discussion and, if necessary, consulting another author (MSL).

### Data synthesis

Chi-square test was used to compare the response rates. The relative risk (RR), mean difference and 95% confidence intervals (95%CIs) from each study were estimated with Review Manager (RevMan) Version 5.0 for Windows (Nordic Cochrane Center, Denmark).

## Results

### Study description

**Figure 1:**
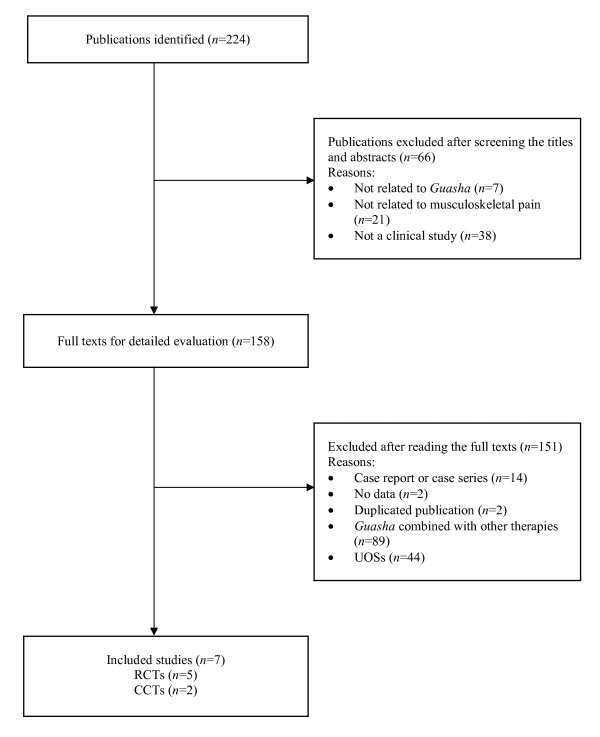
**Flowchart of the selection process for the trials**. RCT: randomized controlled trial. CCT: controlled clinical trial. UOS: uncontrolled observational study.

The literature search found 224 articles, of which 217 were excluded after the full texts were retrieved (Figure 1). A total of 151 studies were excluded because they did not have control (*n *= 44) or they were part of a complex treatment or concomitant use of other therapies (*n *= 89). Five randomized controlled trials (RCTs) [5-9] and two controlled clinical trials (CCTs) [10,11] fulfilled the inclusion criteria (Table 1). All included studies were conducted in China, including treatment for fibromyalgia (1 trial) [5], neck stiffness (1 trial) [6], cervical spondylosis (3 trials) [7,8,10], scapulohumeral periarthritis (1 trial) [9] and lumbar disc herniation (1 trial) [11]. These studies were divided into four categories: (1) recovery, (2) marked improvement, (3) improvement and (4) no change. The sample sizes ranged between 60 and 240.

### Assessment of risk of bias

All of the included studies (five RCTs and two CCTs) had risks of performance bias, attrition bias and detection bias. None of these studies reported randomization methods or allocation concealment or the blinding of the outcome assessors. Dropouts and withdrawals were not mentioned in these studies.

### Outcomes

One RCT comparing *Guasha *with acupuncture reported significantly favorable effects of *Guasha *on pain and the number of pain points in fibromyalgia patients [5]. Another RCT comparing *Guasha *with massage and electric current therapy did not show beneficial effects of *Guasha *in patients with neck stiffness [6]. Two other RCTs comparing *Guasha *with herbal injection in patients with cervical spondylosis [7] or no treatment did not show favorable effects of *Guasha *[8]. The last RCT comparing *Guasha *with acupuncture in patients with scapulohumeral periarthritis reported that *Guasha *was superior in recovery rate [9].

One CCT comparing effects of *Guasha *in patients with cervical spondylosis with acupuncture found favorable effects of *Guasha *on the recovery rate in patients [10]. Another CCT comparing effects of *Guasha *in patients with lumbar disc herniation with acupuncture plus moxibustion did not find favorable effects of *Guasha *[11].

In all seven studies, no adverse events were reported.

## Discussion

Low-quality trials are more likely to overestimate effect sizes [12]. In the case of *Guasha*, few rigorous trials have tested the effects of *Guasha *on MS pain and evidence from the included studiesis limited. In terms of sequence generation, blinding, incomplete outcome measures and allocation concealment, all of the included studies had a high risk of biases. None of the studies reported allocation concealment.

*Guasha *was compared with massage or electric current therapy [6], herbal injection [7], no treatment [8] or acupuncture [5,9-11]. While beneficial effects of *Guasha *compared to acupuncture were found in two trials [5,9], such trials comparing the effects of *Guasha *with another unproved treatment are not informative. One RCT failed to show that *Guasha *is better than massage or electric current therapy. The other RCT failed to show favorable effects of *Guasha *when compared to no treatment in patients with cervical spondylosis [8]. This may suggest that the effects of *Guasha *are non-specific. Controlled trials indicated that *Guasha *reduced MS pain in cervical spondylosis patients but not in patients with lumbar disc herniation [10,11]. All of the included trials failed to report details of statistical analysis; thus, it is difficult to interpret the results. Although three studies reported favorable effects of *Guasha *[8,10,11], our re-analysis failed to show the claimed effectiveness in pain relief (Table 1).

Our review has a number of important limitations. Although strong efforts were made to retrieve all controlled clinical trials on the subject, we are not absolutely certain that we succeeded in doing so. Biases in publishing and reporting are possible [13,14]. It is also possible that negative RCTs remain unpublished and thus the overall picture may be even less positive.

Future RCTs of *Guasha *on pain management should adhere to accepted standards of trial methodology and consider combined use of *Guasha *and other therapies. Sufficient sample sizes, validated outcome measures and an adequate placebo procedure for *Guasha *are necessary in further research.

## Conclusion

Current evidence is insufficient to show that *Guasha *is effective in pain management. Further RCTs are warranted and methodological quality should be improved.

## Competing interests

The authors declare that they have no competing interests.

## Authors' contributions

MSL and JIK conceived the study design. MSL, TYC and JIK searched and selected the trials, extracted, analyzed and interpreted the data. MSL and TYC drafted the manuscript. SMC helped with the study design and critically reviewed the manuscript. All authors read and approved the final version of the manuscript.
